# Hydrodynamic Selection of Polymorphic Assembly Pathways in Plant Protein Amyloid Fibrils

**DOI:** 10.1002/advs.78458

**Published:** 2026-10-26

**Authors:** Zejian Xu, Pengfei Yang, Meng Li, Zichen Cao, Tian Lan, Yan Zhang, Weibiao Zhou, Lianzhou Jiang, Xiaonan Sui

**Affiliations:** ^1^ College of Food Science Northeast Agricultural University Harbin People's Republic of China; ^2^ Heilongjiang Joint Laboratory of Plant‐Based Food Science (International Cooperation) Harbin People's Republic of China; ^3^ College of Horticulture and Landscape Architecture Northeast Agricultural University Harbin People's Republic of China; ^4^ Department of Food Science and Technology National University of Singapore Singapore Singapore

**Keywords:** amyloid fibrils, hydrodynamic selection, plant protein, polymorphic assembly pathways

## Abstract

Plant protein amyloid fibrils are universally assembled in liquid environments, yet their formation is often analyzed without explicitly considering the role of fluid motion. In practice, as a typical model system, soy protein amyloid fibrils (SAFs) are generated under dynamic hydrodynamic conditions involving shear and vortex transport. How such flow fields regulate fibril growth pathways and give rise to polymorphic assembly remains largely unexplored. Here, we elucidate the hydrodynamic selection of SAFs assembly pathways through nanoscale structural characterization combined with flow‐field analysis. Atomic force microscopy (AFM) identifies six distinct fibril morphologies, including short‐curved, javelin‐like, loop‐like, twisted ribbon, helical ribbon, and rod‐like structures. By integrating AFM observations with particle image velocimetry (PIV) results, we propose a drafting, kissing, and docking (DKD) dynamic as a plausible hydrodynamic model for understanding polymorphic fibril assembly. Computational fluid dynamics (CFD) simulations further reproduce key characteristics of the experimentally observed flow fields and provide hydrodynamic support for the proposed interpretation of fibrillation pathways. These results demonstrate that an in‐depth understanding of hydrodynamic selection behavior in polymorphic assembly pathways is important to pave the way for production of tailormade amyloid materials.

## Graphical abstract

Hydrodynamic conditions are revealed to govern polymorphic assembly of soy protein amyloid fibrils. Distinct flow‐induced drafting, kissing, and docking dynamics direct fibril growth into multiple morphologies, linking fluid mechanics with nanoscale assembly and enabling rational design of functional amyloid materials.

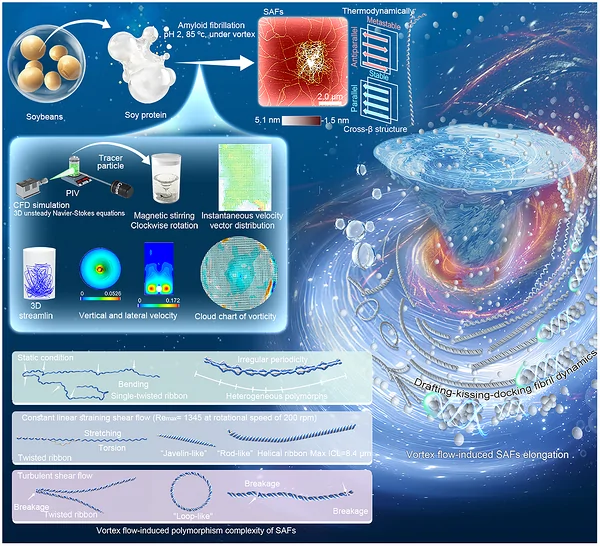

## 1 Introduction

Amyloid fibrils characterized by a conserved cross‐β structure were originally identified in association with human pathologies [1, 2, 3]. By contrast, amyloid fibrils derived from plant proteins are non‐toxic and nutritionally beneficial [4, 5, 6, 7], and have emerged as versatile building blocks with a broad spectrum of techno‐functional properties, including antioxidant activity, emulsification, gelation, foaming, and bioactive nutrient delivery. Recent studies further highlight the potential of plant‐ and food‐derived amyloid fibrils as sustainable functional materials and as platforms for nutrient delivery and gastrointestinal health applications [8, 9]. Such functional diversity is intimately linked to the pronounced polymorphic complexity of fibril structure, which is highly sensitive to fibrillation conditions. Previous studies, including our own, have demonstrated that fibril formation and morphology can be modulated by protein concentration [10], metal ion coordination [11], homologous seeding [12], enzymatic hydrolysis [13], and thermal pretreatments [14]. Importantly, the results of these works have provided common and valid evidence that the hydrogel performance is profoundly governed by the morphologies of fibrillar building blocks.

Protein fibrillation, however, inherently occurs in liquid environments where proteins are exposed to non‐equilibrium hydrodynamic conditions. A range of flow geometries, such as Couette [15] and extensional flows [16], have been shown to induce protein unfolding, aggregation, and amyloid formation. In practical plant protein fibrillation systems, acidic heating under free‐surface vortex flow fields generated by agitation represents one of the most prevalent yet least mechanistically understood conditions. Agitation and mechanical shear are known to accelerate thermal unfolding, shorten fibrillation lag phases [17, 18], and promote the formation of shorter fibrils by facilitating fibril fragmentation and secondary nucleation [19, 20]. Moreover, shear‐mediated detachment of nucleated species from fibril surfaces [21] provides a plausible origin for polymorphic diversification. Despite these observations, how hydrodynamic flow fields regulate fibril growth pathways and give rise to polymorphic assembly remains unclear. In addition to mechanistic uncertainty, conventional acidic heating–based fibrillation strategies suffer from long processing times, high energy consumption, and limited conversion efficiencies. Reported conversion rates for rice protein (60.32%) [22], β‐lactoglobulin (45.00%) [23], and soy protein (82.00%) [14] amyloid fibrils are typically below quantitative yields. Elucidating the hydrodynamic roles that govern polymorphic assembly pathways in vortex flow fields is therefore critical for achieving rapid, energy‐efficient, and controllable formation of target SAFs.

Here, we systematically investigate hydrodynamic flow field–regulated fibrillation pathways of soy proteins in free‐surface vortex flows generated by magnetic stirring. By integrating AFM with PIV and CFD simulations incorporating the apparent viscosity of the protein system, we establish a mechanistic framework linking vortex flow fields to SAFs polymorphism and assembly pathways.

## 2 Results and Discussion

### 2.1 Polymorphic Structures of SAFs

Soy protein readily self‐assembles into amyloid fibrils exhibiting pronounced polymorphic diversity, as reported previously [10, 11, 24, 25, 26]. However, fibrillation invariably proceeds under hydrodynamic conditions, and the role of flow fields in directing fibril assembly remains insufficiently understood. To establish a direct link between fluid dynamics and SAFs polymorphism, we investigated fibril formation under free‐surface vortex flows generated by magnetic stirring at pH 2 and 85°C (Figure 1a). The CFD simulations were performed to resolve the shear flow fields generated at rotational speeds ranging from 0 to 1200 rpm, and representative three‐dimensional streamlines are shown in Figure 1a. Unlike microfluidic [27, 28] or Couette flow [15] systems that allow precise control of shear rates, the complex hydrodynamic conditions in free‐surface vortex flows were quantitatively characterized using the Reynolds number (*Re*). *Re* values were calculated using the equation *Re* = *d𝑢ρ/µ*, from experimentally determined dynamic viscosity (*µ*) obtained by oscillatory rheological test, the equivalent pipe diameter (*d*), the fluid density (*ρ*), and the fluid velocity (*𝑢*) obtained by PIV. Considering the complex geometry of the free‐surface vortex flow, *Re*_max_ was used here as a global comparative descriptor of hydrodynamic intensity among different rotational speeds rather than as a strict criterion for flow‐regime classification. As summarized in Figure 1a, *Re*_max_ increased from ∼1345 at 200 rpm to > 10 000 at 1200 rpm, indicating a progressive increase in overall hydrodynamic intensity with increasing rotational speed. Under static conditions (0 rpm), soy protein isolate (SPI) molecules undergo sedimentation‐induced motion [29], vertical thermal convection driven by temperature gradients at both liquid–liquid and air–liquid interfaces, as well as irregular Brownian motion. In contrast, with increasing rotational speed and hydrodynamic stress, SPI molecules exhibit progressively enhanced mobility and collision frequency within the fibrillation system.

**FIGURE 1:**
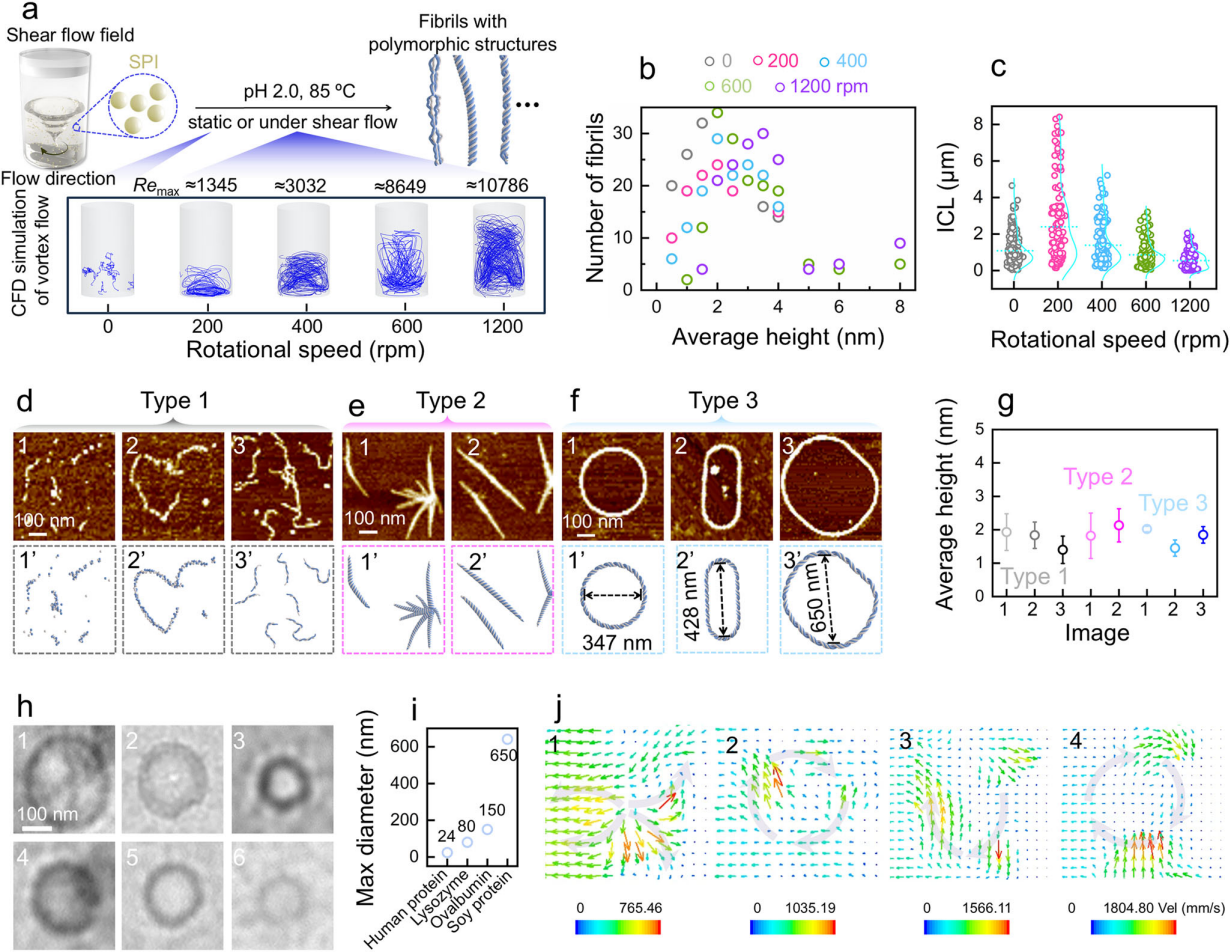
Polymorphic structures of SAFs. (a) The schematic diagram of polymorphic fibril formation under free‐surface vortex flows generated by magnetic stirring at pH 2 and 85°C. Three‐dimensional streamlines obtained by CFD simulation at the bottom of the picture resolve the shear flow fields generated at rotational speeds ranging from 0 to 1200 rpm. (b) The quantitative distribution of SAFs with distinguished average height (0.5–8 nm). (c) The ICL distributions of SAFs obtained at different rotational speeds. (d–f) Representative AFM images (scale bars were 100 nm) of SAFs polymorphic structures (type 1–3), the nether are the corresponding three‐dimensional schematic reconstructions. (g) Average heights of SAFs (type 1–3). (h) TEM images of the loop‐like SAFs obtained at high rotational speed of 600 and 1200 rpm. (i) The maximum diameter of annular amyloid fibrils derived from human protein, lysozyme, ovalbumin, and soy protein. (j) The local flow conditions at rotational speed of 600 rpm using the PIV method.

To systematically evaluate SAFs polymorphism during fibrillation (acidic heating for 0–48 h), we acquired AFM images of diluted solutions under various shear flow conditions. From each condition, we randomly selected 150 individual fibrils whose internal contour lengths (ICL) exceeded 10 nm. The analysis of average height and ICL distributions was performed using FiberApp software [30]. The average fibril height, a topological indicator of fibril maturity, is typically ∼4 nm for mature SAFs. As shown in Figure 1b, under static conditions, most SAFs exhibited average heights ranging from 0.5 to 2 nm, whereas those formed under shear flow conditions (200–1200 rpm) showed average heights mainly within 1.5–4 nm, indicating a pronounced lag effect of static conditions on fibrillation [10, 25]. In contrast, excessive hydrodynamic shear led to progressively shorter fibrils: the mean ICL decreased from ∼2398 nm at 200 rpm to ∼548 nm at 1200 rpm (Figure 1c), consistent with shear‐induced fragmentation limiting fibril elongation [29, 31].

Representative AFM images of SAFs polymorphic structures are presented in Figures 1d–f and 2a–c, accompanied by three‐dimensional schematic reconstructions that illustrate their structural conformations. Consistent with our previous classifications [10, 25], SAFs were broadly divided into short‐curved fibrils and long fibrils. Short‐curved fibrils (type 1; Figure 1d), with average heights of approximately 1–2 nm, were ubiquitously observed under all flow conditions. In contrast to previous works, long SAFs were first classified into five distinct structural types: javelin‐like fibrils (type 2; Figure 1e and Figure S1a), loop‐like fibrils (type 3; Figure 1f and Figure S1b,c), twisted ribbon fibrils (type 4), helical ribbon fibrils (type 5), and rod‐like (nanotube‐like) fibrils (type 6). Javelin‐like fibrils exhibited tapered ends with average heights close to 2 nm and average pitch of 35.2 nm (Figure 1e). This fibril type was primarily observed under static or relatively lower hydrodynamic stress conditions. Their absence under higher hydrodynamic stress conditions suggests that strong shear accelerates the detachment of nucleated species from aggregated cores, thereby hindering cooperative fibril elongation (Figure 1e and Figure S2a). Loop‐like fibrils displayed average heights of approximately 2 nm, average pitch of 20.7 nm, and inner diameters ranging from 80 to 650 nm (Figure 1f,h). These annular structures were observed exclusively at high rotational speeds of 600 and 1200 rpm. For comparison, annular amyloid fibrils derived from human proteins typically exhibit diameters of 6–24 nm [32, 33], while those formed from lysozyme [34] and ovalbumin [35] reach maximum diameters of ∼80 and ∼150 nm, respectively, which are substantially smaller than those of SAFs (Figure 1i). Notably, the curvature distribution of loop‐like SAFs was often heterogeneous, occasionally forming square or rectangular geometries (Figure 1f,h and Figure S1b,c). PIV analysis at 600 rpm (*Re* ≈ 8649) revealed widespread annular vortex flows, U‐shaped recirculation regions, and jet‐like flows (Figure 1j), suggesting that the geometric diversity of local shear flow influences fibril curvature distribution. This geometric diversity of flow patterns is therefore closely associated with the broad curvature distribution observed for loop‐like SAFs. In addition, sustained energy input under higher hydrodynamic stress conditions likely compensates for the enthalpic cost of fibril bending and end‐to‐end closure [36]. Despite this energetic compensation, loop‐like SAFs were observed only at high rotational speeds (600 and 1200 rpm), suggesting that these annular fibrils represent a shear flow‐dependent polymorphic state associated with strong hydrodynamic shear forces (HSFs). Their near absence at lower rotational speeds further supports the notion that elevated shear and complex vortex flow fields are prerequisite, yet insufficient, conditions for stabilizing loop‐like SAFs. Twisted ribbon (type 4) and helical ribbon (type 5) fibrils, with average heights ranging from 0.5 to 4 nm and average pitch ranging from 20 to 70 nm (Figure 2a,b,d), were observed under both static and shear flow conditions. The longest helical ribbon fibrils obtained at 200 rpm exhibited ICL values up to 8.4 µm (Figure S2b). In contrast, rod‐like fibrils (type 6) displayed larger average heights of 5–8 nm (Figure 2c,e) and lacked clear periodic features (average pitch < 20 nm), indicating a distinct assembly pathway. While fibril types 1, 4, and 5 have been reported previously for plant protein–derived amyloid fibrils [7, 37, 38], the present results highlight that shear flow fields are closely associated with shear flow‐dependent occurrence of less commonly observed SAF polymorphs, including types 2, 3, and 6.

**FIGURE 2:**
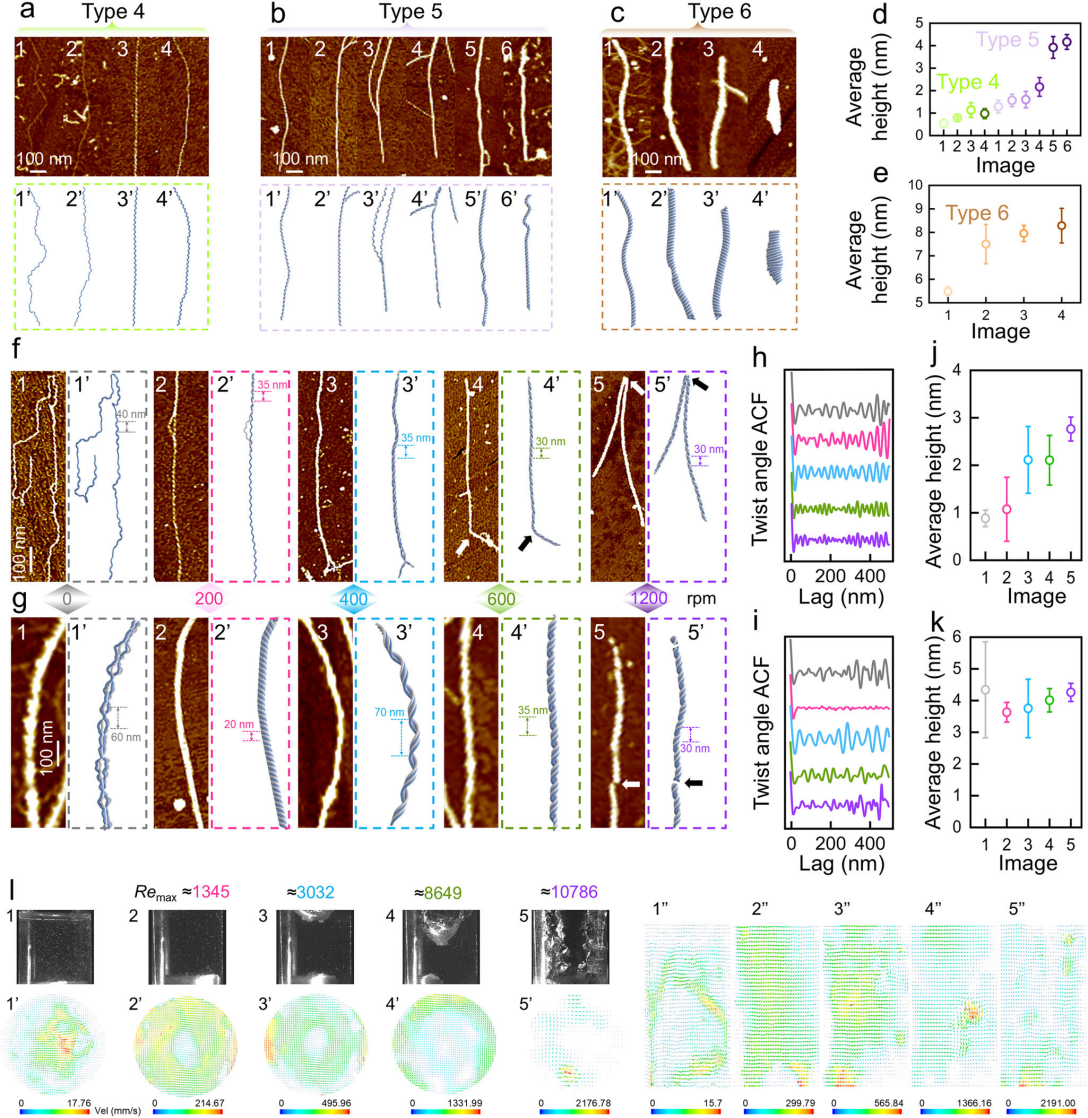
Morphological diversity and topological rearrangements of long SAFs. The predominant long SAFs and their topological rearrangements. (a–c) Representative AFM images (scale bars were 100 nm) of long SAFs polymorphic structures (type 4–6), the nether are the corresponding three‐dimensional schematic reconstructions. (d,e) Average heights of long SAFs (type 4–6). (f,g) Flow‐dependent polymorphic evolution of long SAFs obtained at acidic heating time of (f) 12, (g) 48 h and different rotational speeds (scale bars: 100 nm). (f1’–5’,g1’–5’) Three‐dimensional schematic reconstructions of the targeted fibrils with labeled periodicity. (h,i) The twist angle autocorrelation function (ACF) profiles of the target fibrils. (j,k) The average heights of the target fibrils. (l) PIV measurements reveal the velocity distribution in the fluid field. (l1–l5) The fluid flow visualization under various flow conditions. (l1’–l5’, l1″–l5″) The instantaneous velocity vector distribution of the fluid flow in both vertical and lateral directions. The unsatisfactory results of l5’ and l5″ were due to the poor tracing particle photography caused by the reflection effect produced by high‐speed fluid flow.

### 2.2 Topological Rearrangements of SAFs

To determine how shear flow fields actively reshape the fibrillar aggregation pathway of soy protein, we focused on the topological rearrangements of long SAFs during fibrillation. AFM analysis of diluted fibril‐forming solutions generated under different shear flow conditions (Figure S3) revealed pronounced, flow‐dependent polymorphic evolution. Long fibrils collected after 12 and 48 h of acidic heating (Figure 2f1–5,g1–5) were quantitatively analyzed using FiberApp software, allowing reconstruction of fibril periodicity, height, and three‐dimensional topology (Figure 2f1’–5’,g1’–5’ and Figure S4). Twist‐angle autocorrelation analysis further resolved pitch sizes (Figure 2h,i), enabling direct comparison of fibril maturation across hydrodynamic regimes. The average heights of each fibril were also given in Figure 2j,k. Under shear flow, protein monomers and oligomers undergo longitudinal association and alignment driven by stretching events imposed by HSFs, which reduce configurational entropy and bias assembly toward anisotropic growth. This stretching‐mediated alignment constitutes a critical physical basis for shear‐induced pathway selection [15, 39, 40]. Under static conditions, single twisted ribbon fibrils exhibited low average heights (< 1 nm), large periodicity fluctuations, and frequent accidental bending along their contour length (Figure 2a1,f,h,j and Figure S5). In parallel, long rigid fibrils with average heights exceeding 4 nm displayed irregular and poorly defined periodicity (Figure 2g,i,k). PIV measurements revealed that, in the absence of imposed shear, protein species were mainly transported by weak convective flows induced by vertical thermal gradients (Figure 2l1’,1″). Although such convective flows have been reported to support amyloid growth [41], their mechanical energy input is substantially lower than that of shear flow fields. Consequently, static conditions favor multiple competing aggregation pathways, resulting in enhanced polymorphic heterogeneity rather than topological convergence. This low hydrodynamic stress environment favors the emergence of structurally distinct polymorphs, including single twisted ribbon fibrils and long rigid fibrils with irregular periodicity, consistent with previous observations that reduced environmental stress enhances pathway multiplicity and polymorphic heterogeneity [27, 42, 43].

Quantitative analysis of long fibrils obtained after 12 h of acidic heating revealed a clear shear‐dependent maturation trend. Increasing rotational speed led to higher average fibril height (Figure 2j) and reduced periodicity (Figure 2h), indicating enhanced structural rigidity within a fixed time window. Previous observations suggested that shear flow fields bias nucleation and fusion events, thereby promoting fibril growth kinetics [36] and driving structural consolidation. However, this maturation enhancement was not monotonic. When rotational speeds exceeded 200 rpm (*Re* ≈ 1345), periodicity decreased and partial fibril breakage was observed (Figure 2f4,5,g5), indicating the onset of structural destabilization. PIV measurements provide a mechanistic explanation for this transition. At moderate rotational speeds, instantaneous velocity vector distributions in the vertical direction (Figure 2l2’) revealed a well‐aligned laminar structure near the vortex core, characterized by minimal interaction between adjacent fluid layers. As rotational speed increased, this laminar coherence deteriorated rapidly, a phenomenon corroborated by velocity vector distributions in the lateral direction (Figure 2l3″–5″). These results indicate that fibril maturation is optimized within a defined hydrodynamic regime rather than under maximal shear input. A uniform and constant laminar shear field provides sufficient mechanical bias to promote directional association and topological convergence, while avoiding the destabilizing effects of excessive flow heterogeneity. We therefore identify this laminar straining regime as the optimal window for the formation of mature long rigid SAFs. To elucidate the significance of the laminar straining regime, we performed frequency sweep rheology test on fibril hydrogels obtained at 200 rpm (*Re*_max_ ≈ 1345) and 600 rpm (*Re*_max_ ≈ 8649) following acidic heating for 6, 12, and 24 h (Figure S6), with corresponding fibril morphologies displayed in Figure S3. The rheological results indicated that long fibrils, formed under a constant laminar shear field, enhanced the construction of hydrogel networks.

At the early stages of fibrillation, assembly pathway diversity is already encoded at the level of the nuclei. As shown in Figure 3a, nuclei display a broad height distribution ranging from 0.6 to 8.9 nm, and three‐dimensional AFM reconstructions reveal pronounced topological heterogeneity rather than idealized spherical geometries, in agreement with previous reports [44]. Notably, shear regulates the interaction dynamics among nuclei. The templating propensity of associating nuclei [45] is governed by collision events, which under static conditions occur stochastically within the convective flow field. We therefore propose that chaotic nucleation and random collision events initiate diverse polymorphic templating motifs, which are subsequently stabilized through steric zipper interactions [46]. In the absence of directional shear selection, this process gives rise to long rigid fibrils with irregular periodicity, reflecting the structural memory of heterogeneous nucleus topologies. Furthermore, vortex‐type flow fields, including free‐surface vortex flow, micro‐vortex flow [28], and Taylor vortex flow [47], share a common hydrodynamic feature: their capacity to induce chiral symmetry breaking in nominally achiral systems. This raises a central question in SAFs fibrillation—whether hydrodynamic shear not only accelerates assembly but also regulates the handedness features of protofilaments during polymorphic transitions. As shown in Figure 3b, the handedness distribution and average pitch values of protofilaments are strongly dependent on rotational speed. Quantitatively, left‐handed protofilaments predominate overall, consistent with the intrinsic left‐handedness of β‐sheet structures [45]. Under static conditions, most protofilaments exhibit either left‐handedness (H_L_) or no defined handedness (H_N_), with average heights confined to ∼1–2 nm, indicative of early‐stage assembly. Upon application of moderate shear (200 rpm), both H_L_ and right‐handed (H_R_) protofilaments with average heights spanning 1–4 nm become dominant, reflecting enhanced structural diversification and progression toward higher‐order assembly. As rotational speed increases further (600 and 1200 rpm), protofilaments progressively lose well‐defined handedness and increasingly adopt non‐handed morphologies. Notably, the average heights of H_L_ and H_R_ protofilaments increase markedly between 200 and 600 rpm, except for those near ∼1 nm‐approaching ∼4 nm, which we previously identified as a critical indicator of SAFs maturation. These results indicate that shear flow fields do not simply bias one handedness over another; rather, they modulate the chiral phase space of protofilaments in a maturation‐dependent manner. Moderate laminar shear promotes the emergence and amplification of well‐defined chiral species associated with structural consolidation, whereas excessive shear drives the system toward chirality attenuation and topological reconfiguration. Collectively, this shear‐dependent handedness features distribution provides further evidence that shear flow fields function as pathway selectors, orchestrating not only fibril growth but also hierarchical chiral organization.

**FIGURE 3:**
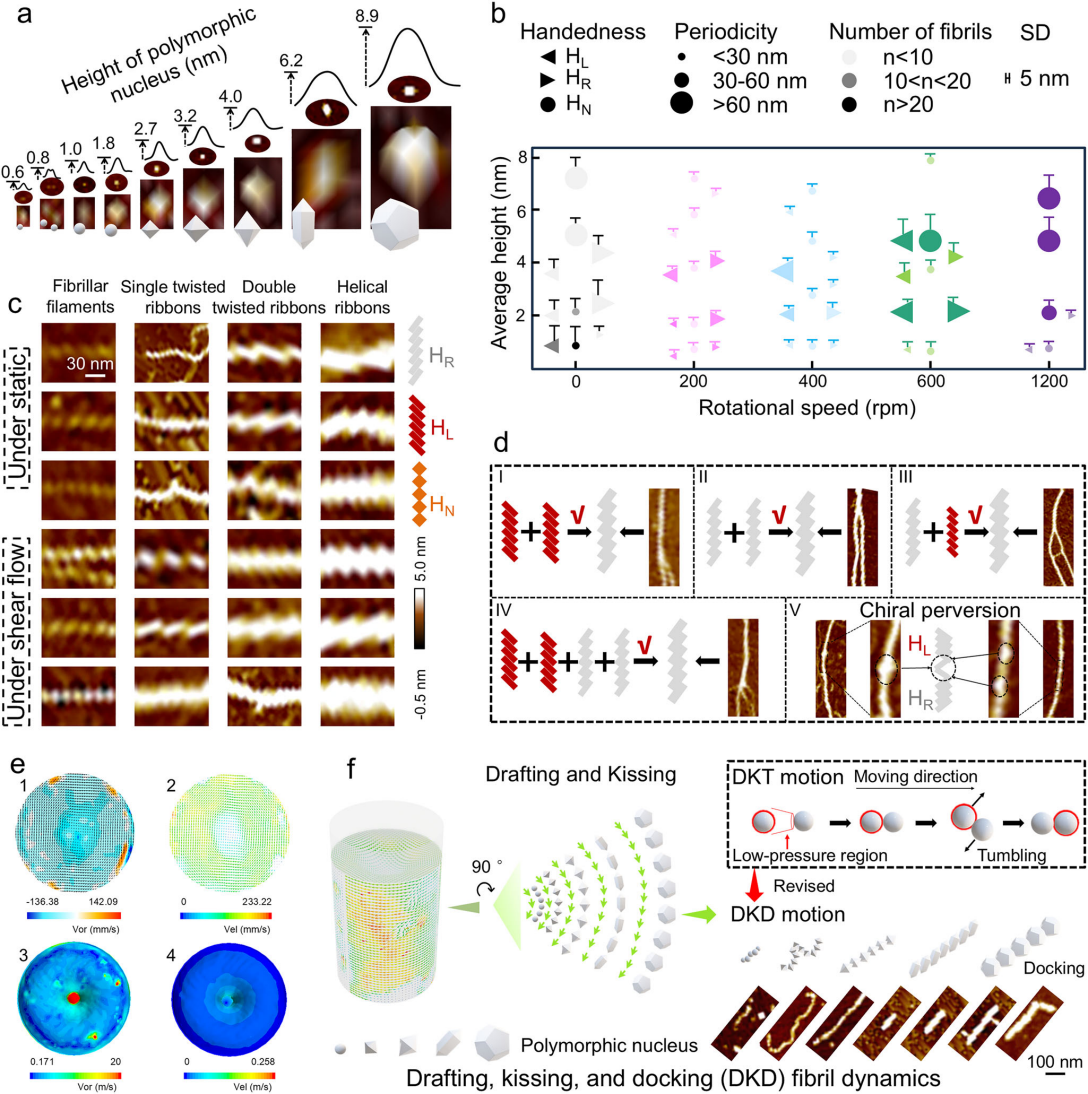
Topological rearrangements of SAFs and the fluid motion behaviors. (a) AFM analysis of the variations in the height of polymorphic nucleus, which range from 0.6 to 8.9 nm. The magnified three‐dimensional top views and models were positioned below the corresponding nucleus. (b) The handedness distribution and average pitch values of protofilaments obtained at various rotational speeds. (c) The magnified AFM images of the representative fibrillar filaments, single twisted ribbons, double twisted ribbons and helical ribbons (scale bars: 30 nm). (d) The schematic conversion maps summarizing the chirality transition routes observed during protofilament polymorphic evolution. (e) PIV and CFD simulations results of the (1, 3) vorticity and (2, 4) velocity field of the free‐surface vortex flow generated at rotational speed of 200 rpm in the vertical plane. (f) The schematic diagram of drafting, kissing, and docking (DKD) fibril dynamics under shear flow fields.

Although a higher maturation level is generally associated with reduced average periodicity [10, 12, 48], our results reveal that this relationship becomes non‐monotonic under increasing shear input. Specifically, the average pitch values of fibrils formed at 200, 400, 600, and 1200 rpm were 20, 70, 35, and 30 nm, respectively (Figure 2g2’–5’), indicating that shear does not simply compress helical pitch in a linear fashion. Instead, the chirality‐periodicity coupling appears to be dynamically reshaped by hydrodynamic conditions. To further elucidate this behavior, magnified AFM images of representative fibrillar filaments, single twisted ribbons, double twisted ribbons, and helical ribbons obtained under various shear flow conditions are shown in Figure 3c. Two critical features emerge. First, for single twisted ribbons, the average pitch value under shear flow is nearly threefold greater than that under static conditions, independent of handedness. This suggests that moderate shear introduces mechanical strain that elongates the helical pitch rather than tightening it. Second, fluctuations in periodicity among different protofilament species are markedly reduced under shear flow compared to static conditions, implying that shear flow fields constrain structural heterogeneity while modulating torsional geometry. Previous studies have proposed that hierarchical multistranded self‐assembly into helical aggregates arises from competition between the intrinsic chirality of peptide building blocks [49]. Our observations suggest that HSFs perturb this competitive balance by injecting mechanical energy, thereby altering strain distribution across assembling protofilaments. In this framework, shear acts not merely as a kinetic accelerator but as a mechanical regulator that redistributes torsional stress, reshaping polymorphic outcomes. At the highest rotational speed (1200 rpm), most protofilaments exhibit no discernible handedness and adopt nanotube‐like morphologies (Figure 3b). According to theoretical models describing the energy evolution of polymorphic transitions toward nanotube fibrils [50], helical ribbons can further lower their free energy by minimizing twist and releasing edge‐associated line tension. The prevalence of nanotube‐like structures under high shear flow therefore suggests that excessive hydrodynamic input drives the system toward a distinct thermodynamic basin characterized by reduced torsional frustration. Together, these observations indicate that shear flow fields reshape strain distribution across assembling protofilaments, biasing polymorphic transitions toward lower‐energy configurations.

During hierarchical amyloid fibrillization, mass production and polymorphic transitions are governed by primary nucleation, elongation, and secondary nucleation [21, 51, 52], while the ultimate maturation state is determined by the intertwining pathways of protofilaments [45, 48]. To dissect how these intertwining processes evolve under shear, we constructed schematic conversion maps summarizing the chirality transition routes observed during protofilament polymorphic evolution (Figure 3d). Strikingly, four possible conversion routes converging toward a mature right‐handed twisted ribbon were identified, characterized by an average height of ∼4 nm and average pitch value of ∼30 nm. These routes may involve: (I) two left‐handed thinner protofilaments; (II) two right‐handed thinner protofilaments; (III) one left‐handed and one right‐handed thinner protofilament; and (IV) the coalescence of two left‐handed and two right‐handed thinner protofilaments. To our knowledge, this work provides the first identification of four possible conversion routes in soy protein amyloid fibrillation.

For other amyloid systems, routes I, III, and IV are consistent with previous reports [48, 53], supporting the generality of hierarchical protofilament intertwining as a dominant maturation pathway. Route II, however, challenges conventional chirality conversion rules, as fibrillar aggregates are typically expected to exhibit handedness opposite to that of their constituent protofilaments [53, 54]. The emergence of this route suggests that external mechanical input may override intrinsic chirality constraints. Analogously, in textile engineering, torque‐balanced fibrillar assemblies can be produced by heterochiral plied twistrons, resembling route I, whereas assemblies generated via routes II‐IV generally display lower resistance to untwisting [55, 56, 57]. This comparison implies that torsional torque introduced by HSFs may actively reshape the energetic hierarchy among competing intertwining pathways. Beyond these four routes, we further observed a previously unreported scenario (route V) in which right‐ and left‐handed features coexist along the same fibril, accompanied by pronounced periodic height fluctuations. This phenomenon reflects chirality perversion driven by intrinsic curvature and constrained by topological conservation laws [58, 59, 60, 61], adding another layer of polymorphic complexity. Taken together, these findings suggest that shear flow fields not only accelerate fibrillation but also expand and reorganize the topological conversion landscape of protofilaments. Whether shear fundamentally governs chirality conversion or merely biases an intrinsically accessible pathway space remains an open mechanistic question, warranting further investigation.

In light of the foregoing structural analyses, it becomes essential to explicitly bridge polymorphic complexity with hydrodynamic behavior. The velocity vector distributions in the vertical and lateral directions (PIV Movies S1–S10) provide direct visualization of flow orientation and fluctuation intensity, while the averaged velocity cloud maps and statistical distributions (Figure S7) quantify the underlying dynamics. Across all conditions, the most pronounced distinction resides in the population and stability of laminar flow states. At 200 rpm (*Re* ≈ 1345), the free‐surface vortex flow exhibits a highly concentrated vortex core with smooth concentric spiral streamlines in the vertical plane (Figure 3e). The associated vorticity and velocity fields indicate a coherent laminar regime characterized by persistent linear straining. Such constant straining shear flow has been shown to reinforce directional nucleation and aggregation of protein solutes [47]. Notably, linear shear flow can be decomposed into the superposition of rotational and extensional components [62], implying that fibril dynamics inherently couple tumbling with periodic deformation. This fluid–structure coupling recalls the classical drafting, kissing, and tumbling (DKT) motions observed during gravity‐driven sedimentation in viscous media [63, 64]. In DKT dynamics, a trailing particle accelerates toward a leading particle due to a wake‐induced low‐pressure region, followed by vertical instability and tumbling exchange of positions. Under static conditions, analogous interactions may arise among protein aggregates of comparable size and morphology. However, tumbling inherently disrupts stable end‐to‐end alignment and is therefore incompatible with sustained fibril elongation. We therefore propose a revised dynamical model, termed drafting, kissing, and docking (DKD), to describe a plausible fibril elongation pathway under shear flow conditions (Figure 3f). The key distinction between classical DKT dynamics and the proposed DKD model lies in the post‐contact behavior: DKT involves rotational instability and positional exchange after kissing, whereas DKD favors preservation of axial alignment and subsequent end‐to‐end docking under sustained hydrodynamic orientation. For instance, under vortex‐induced shear flow, the hydrodynamic scenario becomes fundamentally distinct. The presence of torsional flows [65], elongational components [66], and continuous linear straining, together with the polymorphic diversity of nuclei, protofilaments, and fibrils, creates a dynamic environment that favors alignment rather than rotational exchange. In the DKD framework, nucleus species and fibrillar aggregates experience hydrodynamically mediated attraction via localized low‐pressure regions that form at either the leading or trailing edges of moving aggregates, irrespective of whether their velocity exceeds or lags behind the surrounding flow. These pressure gradients promote directional drafting, transient kissing contacts, and ultimately stable docking without tumbling‐induced positional inversion. Such docking events are further stabilized by end‐to‐end hydrophobic interactions [67, 68], long‐range electrostatic repulsion along fibril surfaces [69, 70], and the sustained linear straining shear field, which maintains axial alignment. Collectively, this model suggests that vortex‐induced laminar shear does not merely accelerate collision frequency but fundamentally alters the kinematic pathway of aggregate interaction—from exchange‐dominated DKT motions to alignment‐preserving DKD docking—thereby enabling efficient end‐to‐end elongation and maturation of SAFs.

### 2.3 CFD Simulations and Fibrillation Pathways

To mechanistically support the hydrodynamic interpretations derived from PIV analysis and structural observations, CFD simulations were performed using the re‐normalization group (RNG) k–ε turbulence model [71], which is particularly suitable for capturing vortex‐dominated flows with transitional laminar–turbulent characteristics. The simulations were performed until a statistically steady flow field was obtained. Representative contours of vertical and lateral vorticity, velocity magnitude, and turbulent kinetic energy after establishment of the statistically steady flow field are shown in Figure 4a–c. The complete simulations profiles are provided in Figures S8–S12. The simulations recapitulate the experimentally observed flow features, particularly the persistence of a coherent vortex core and spatial confinement of turbulent fluctuations within the laminar regime at moderate rotational speeds. To further integrate hydrodynamic modeling with fibrillation kinetics, the viscoelastic evolution of fibril‐forming solutions during acidic heating was characterized by frequency sweep rheology. As shown in Figure 4d, the storage modulus (G’) and loss modulus (G″) exhibit markedly distinct temporal trends, reflecting the progressive transition from sol‐like to network‐dominated behavior during fibrillation. In addition, contrast to higher rotational speeds (> 200 rpm), this transition behavior occurred much earlier at lower rotational speeds (≤200 rpm). Moreover, when it occurred at higher rotational speeds (> 200 rpm), its values of G’ and G″ remained substantially lower. These results suggest that moderate hydrodynamic flow fields facilitate the rapid formation of fibril hydrogel networks. This time‐dependent rheological evolution directly affects the flow field through viscosity modulation. Therefore, to enhance the physical fidelity of the CFD framework, experimentally measured apparent viscosity values at different fibrillation stages (Figure 4e) were incorporated into the simulation model. By incorporating experimentally measured, stage‐specific apparent viscosities into separate CFD cases, the simulations provide qualitative insight into the hydrodynamic environments associated with different fibrillation stages.

**FIGURE 4:**
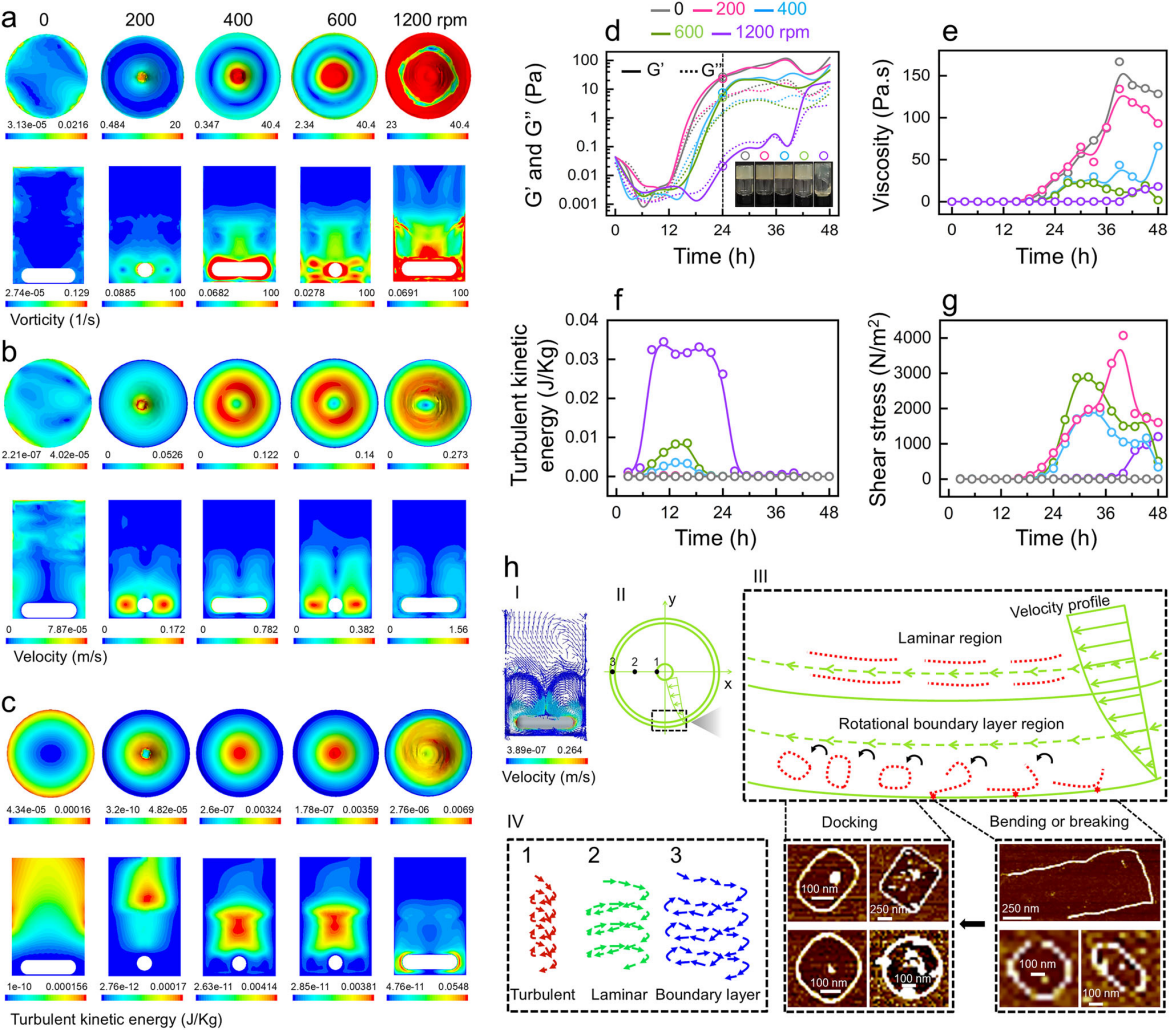
CFD simulations. (a–c) Representative contours of vertical and lateral (a) vorticity, (b) velocity magnitude, and (c) turbulent kinetic energy after establishment of the statistically steady flow field. (d) The storage modulus (G’) and loss modulus (G″) of protein samples along the acidic heating time measured by frequency sweep rheology test at 0.1% strain and angular frequency of 1 rad s^−1^. The inside pictures are fibril hydrogel performance at the acidic heating time of 24 h. (e) The changes in apparent viscosity values of the fibril‐forming solutions as a function of acidic heating time under various flow conditions. (f,g) The changes in (f) turbulent kinetic energy and (g) shear stress as a function of acidic heating time under various flow conditions. (h) The flows in different regions. (h I) The CFD simulations of instantaneous velocity vector distributions. (h II) The flows regions from a top view. (h III) The schematic diagram illustrates that homogeneous linear straining shear gradients promote directional alignment and end‐to‐end elongation of protofilaments, whereas flow in the rotational boundary layer exhibits pronounced shear gradient heterogeneity [71], leading to rotational deformation and curvature redistribution of protofilaments. The AFM images below with different scale bars are the examples of rotational deformation and curvature redistribution of protofilaments. (h IV) The simulated descending torsional flows in different regions.

With continued heating, the dominant driving force shifts to the temperature gradient between the fluid bulk and the container wall, sustaining high levels of vorticity and kinetic energy throughout the system (Figure 4a–c and Figure S8). Concurrently, the apparent viscosity increases dramatically—from ∼0.1 Pa s at ∼15 h to ∼166.7 Pa s at ∼39 h—introducing a progressively stronger coupling between thermal convection and viscous dissipation. The resulting stochastic flow behavior is reflected in the simulated chaotic trajectories of protein particles (Movie S11), consistent with the diversified polymorphic outcomes observed experimentally. At 200 rpm (*Re* ≈ 1345), fluid motion becomes primarily governed by the magnetic stirring rod rather than thermal convection. At early stages of heating, when viscosity remains low, a substantial velocity mismatch exists between the rotating stir bar and the surrounding fluid. This mismatch generates a composite flow field consisting of circumferential rotation and vertical recirculation, with elevated turbulent kinetic energy concentrated in the central annular region (Figure 4a–c and Figure S8). Vorticity is markedly amplified in the vicinity of the stir bar compared to the free surface near the container wall, indicating localized shear intensification. As fibrillation progresses and viscosity increases, the flow field reorganizes. A funnel‐shaped free surface emerges, and the vortex core migrates toward the geometric center of the container. The region of maximum vorticity correspondingly shifts inward, while the mid‐annular region evolves into a mixed circular–vertical flow structure with enhanced velocity magnitude (Figure 4h I). This transition reflects the establishment of a more coherent vortex architecture under increasing viscous constraint. Importantly, distinct hydrodynamic subregions arise within the vortex system: a turbulent vortex core, a surrounding laminar straining region, and a rotational boundary layer (Figure 4hII,IV). Descending torsional flows within these regions introduce spatially heterogeneous shear gradients [72], which differentially influence protofilament dynamics. In the laminar region, homogeneous linear straining shear gradients promote directional alignment and end‐to‐end elongation of protofilaments (Figure 4hIII), consistent with the DKD mechanism proposed above. In contrast, the boundary layer region exhibits pronounced shear gradient heterogeneity [73], leading to rotational deformation and curvature redistribution of protofilaments. Such mechanically induced bending and torsional instability provide a plausible origin for the formation of loop‐like SAFs with variable curvature distributions. To bridge reactor‐scale flow characteristics with the local mechanical environment governing fibril assembly, the local turbulent energy dissipation rate ε (Figure S13), Kolmogorov length scale η (Figure S14), and Kolmogorov time scale τk (Figure S15) were extracted from the CFD simulations. These parameters provide an intermediate‐scale representation of the dissipative and deformation conditions within the reactor. With increasing rotational speed, ε increased, whereas η and τk decreased near the stir bar and boundary‐layer regions, indicating enhanced local dissipation, reduced eddy size, and accelerated deformation dynamics. These hydrodynamic variations agree with the observed association between higher rotational speed and decreased fibril length and modified curvature distribution. Together, these simulations indicate that viscosity‐driven flow reorganization partitions the vortex into mechanically distinct zones, each characterized by a specific shear gradient landscape. This spatial stratification of hydrodynamic forces offers a physical basis for the coexistence of aligned mature fibrils, chiral twisted ribbons, and curvature‐rich loop‐like aggregates within the same reactor environment.

The simulated trajectories of protein particles at 200, 400, 600, and 1200 rpm (Movies S12–S15) reveal progressively intensified hydrodynamic forcing with increasing rotational speed. Although the global vortex architecture remains qualitatively similar to that observed at 200 rpm, the magnitude of vorticity, velocity, and turbulent kinetic energy (Figure 4f) increases systematically as rotational speed rises. This indicates that the system does not transition to a fundamentally different flow topology, but rather experiences continuous amplification of hydrodynamic loading. Such amplification directly alters the mechanical stress spectrum acting on protein aggregates. Increasing rotational speed enhances hydrodynamic drag, torque, and centrifugal forces [74, 75, 76], thereby modulating the balance between axial stretching, rotational deformation, and radial displacement. Under moderate shear, these forces facilitate directional alignment and elongation, consistent with the DKD mechanism described above. However, as turbulent kinetic energy further increases, local shear gradients become more heterogeneous, and transient high‐strain‐rate regions emerge. Comparison of CFD‐derived turbulent kinetic energy and shear stress distributions (Figure 4g) with AFM‐measured fibril morphologies suggests an association between hydrodynamic conditions and fibril length distribution. Elevated turbulent kinetic energy promotes mechanical fragmentation, curvature instability, and structural disorder, leading to shorter and less organized fibrillar assemblies. This turbulence‐dependent shortening effect is in agreement with both AFM observations and PIV‐derived flow characteristics, reinforcing the conclusion that polymorphic outcomes are not solely dictated by biochemical kinetics but are critically regulated by the magnitude and spatial heterogeneity of hydrodynamic stresses.

To mechanistically interpret shear‐dependent polymorphic evolution within the DKD dynamic model, fibrillation pathways were systematically mapped (Figure 5a), and representative AFM‐resolved possible local assembly events are shown in Figure 5b–e. Considering that short‐curved fibrils, long fibrils, and amorphous aggregates constitute the dominant topological classes during amyloid assembly, the observed fibrillation routes can be categorized into four possible principal pathways: pathway 1 (short‐curved fibrils), pathway 2 (long fibrils via monomeric nucleus elongation followed by intertwining), pathway 3 (long fibrils via direct elongation of oligomeric nuclei), and pathway 4 (amorphous aggregation). Across amyloid systems, monomers and oligomers are widely recognized as fundamental building units in hierarchical fibril assembly [77, 78, 79]. During protofilament elongation, both monomeric and oligomeric nuclei may directly incorporate at fibril termini, provided structural compatibility prevents steric hindrance [79, 80, 81]. Importantly, the diameter and mechanical rigidity of elongating protofilaments are largely dictated by the size and packing geometry of the recruited nuclei, thereby influencing subsequent polymorphic outcomes. This distinction underpins the separation of long‐fibril formation into pathway 2 and pathway 3. In pathway 2, monomeric nuclei initially align into thinner fibrillar seeds under HSFs‐induced linear straining, followed by axial elongation and subsequent intertwining into multistranded mature fibrils. The sustained laminar shear field stabilizes directional docking and promotes progressive structural consolidation. In contrast, under static conditions (pathway 2'), stochastic convection perturbs ordered intertwining, leading to single‐twisted ribbon and long‐rigid fibrils with enhanced polymorphic heterogeneity. Pathway 3 originates from oligomeric nuclei that directly transition into thicker fibrillar seeds. These protofilaments elongate without pronounced intermediate intertwining steps and assemble into mature fibrils through continuous axial growth. Under static conditions (pathway 3'), however, twisted and helical ribbons display broader periodicity distributions, reflecting insufficient mechanical bias to constrain torsional fluctuations. These comparisons suggest that HSFs facilitate energy redistribution along assembling protofilaments, effectively lowering barriers associated with edge exposure and promoting edge closure [50]. The enhanced energy migration under shear stabilizes more compact and mature fibrillar conformations with reduced periodicity. For short‐curved fibrils (pathway 1), AFM observations indicate that the primary distinction between static (pathway 1') and shear conditions lies in the degree of entanglement. Static environments favor localized aggregation and cluster formation due to limited directional alignment, whereas shear flow suppresses excessive entanglement through continuous axial straining. A similar trend is observed for amorphous aggregates (pathway 4): aggregates formed under static conditions (pathway 4') are larger and more irregular, while shear flow restricts aggregate size by imposing mechanical fragmentation and alignment constraints. Collectively, these pathway comparisons reveal that shear flow fields do not introduce entirely new assembly routes; rather, they selectively bias energy flow, docking orientation, and strain distribution within existing pathway networks. In this sense, shear flow fields function as a pathway selector, shifting the balance from entanglement‐dominated and torsionally frustrated states toward directionally aligned and structurally consolidated fibrillar architectures.

**FIGURE 5:**
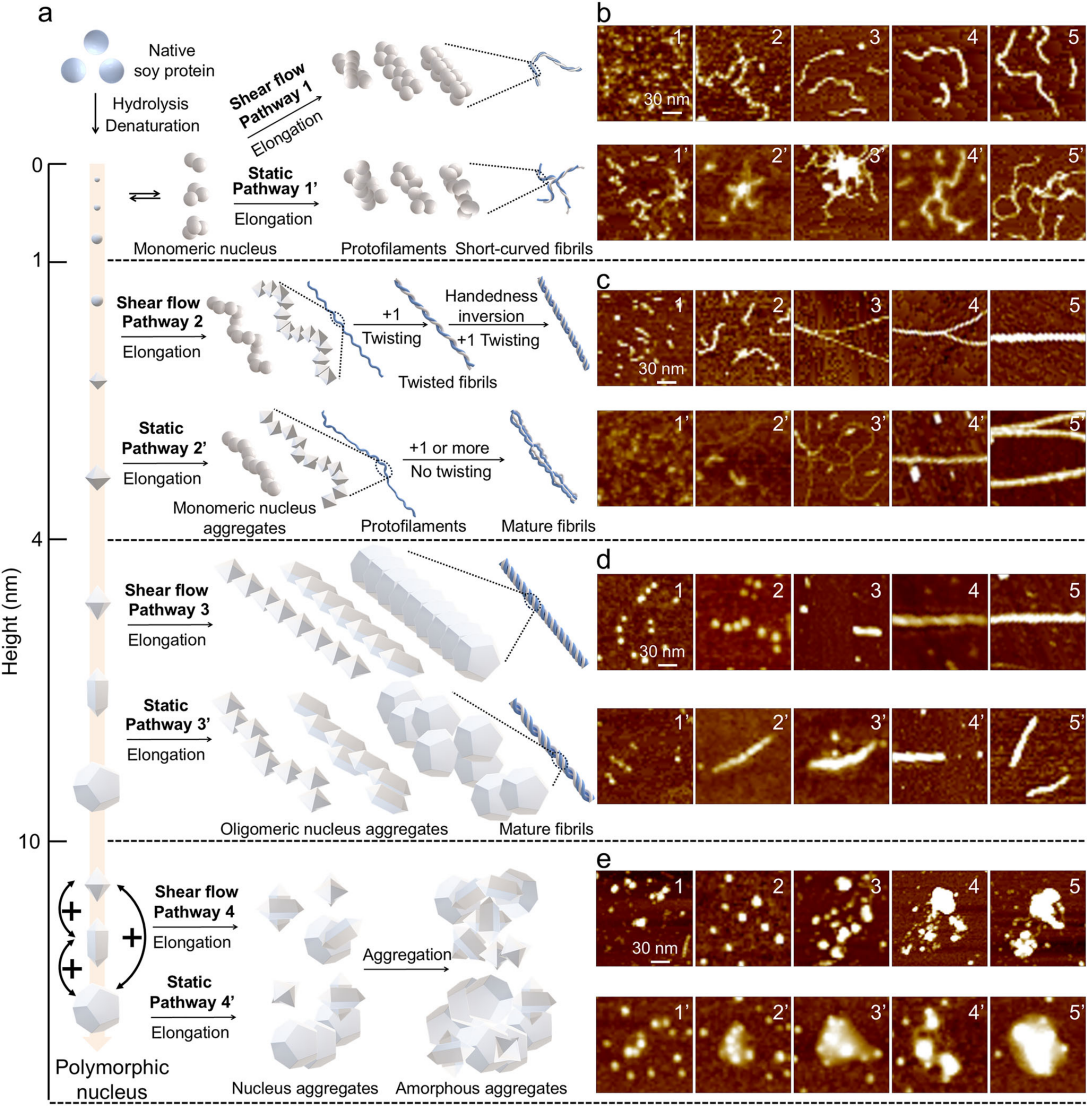
The shear‐dependent fibrillation pathways. (a) The shear‐dependent polymorphic evolution within the DKD dynamic model. (b–e) Representative AFM‐resolved local assembly events (scale bars: 30 nm).

### 2.4 Conformational Remodeling of SAFs

The fibrillation process is fundamentally governed by the thermodynamic drive toward energetically favorable conformational states [23, 82, 83]. To elucidate the role of shear flow fields in conformational remodeling during soy protein fibrillation, the evolution of the Amide I region in FTIR spectra was systematically analyzed. Figure 6a presents the Amide I region (1600–1700 cm^−1^) of soy protein samples obtained under varying rotational speeds during acidic heating (0–48 h). Quantitative secondary structure contents are summarized in Table S1. The fully hydrated soy protein at pH 2 exhibited a dominant absorption peak at 1632.45 cm^−1^, consistent with previous observations [10], indicating extensive acid‐induced unfolding and transition toward β‐sheet‐rich aggregates (β‐sheet content: 56.8%). After 48 h of acidic heating, distinct spectral shifts were observed depending on shear conditions: red shifts under static, 600 , and 1200 rpm conditions, and blue shifts at 200 and 400 rpm, suggesting shear‐dependent modulation of hydrogen bonding and β‐sheet packing. To further resolve these differences, the temporal evolution of secondary structures was quantified (Figure 6b1–4), and the ratio of parallel to antiparallel β‐sheets was calculated (Figure 6c). Given that monomers, oligomers, protofilaments, and mature fibrils possess distinct conformational signatures, the antiparallel β‐sheet is generally considered a dominant structural motif in oligomeric intermediates [84, 85]. Under static conditions, the initial fibril‐forming solution contained 28.16% antiparallel and 28.63% parallel β‐sheets. After 12 h of heating, antiparallel β‐sheets increased to 33.96%, while parallel β‐sheets decreased to 24.84%, reducing the parallel/antiparallel ratio from 1.02 to 0.73 (Figure 6c). This indicates preferential accumulation of metastable antiparallel β‐sheet structures during early aggregation. In contrast, shear flow markedly accelerated β‐sheet formation. The maximum β‐sheet content was reached at 6, 24, 12, and 3 h under 200, 400, 600, and 1200 rpm, respectively (Figure 6b1), demonstrating a clear shear‐dependent acceleration. However, while shear flow fields promoted rapid β‐sheet assembly, the maximum antiparallel β‐sheet contents (35.34%, 34.34%, 34.07%, and 34.17% from 200 to 1200 rpm) did not increase proportionally with shear intensity. This suggests that elevated HSFs facilitate β‐strand formation but disfavor antiparallel inter‐sheet stacking. Parallel β‐sheets exhibited a distinct evolution pattern. Although early‐stage variations differed among rotational speeds, all conditions showed a progressive increase after 12 h (Figure 6b2), coinciding with fibril maturation. This conversion occurred at the expense of α‐helices/random coils and partially β‐turns (Figure 6b3,4), indicating conformational consolidation into ordered cross‐β structures. Previous studies have established that antiparallel β‐sheet fibrils are thermodynamically metastable and can reorganize into parallel β‐sheet architectures through dissolution‐reassembly mechanisms [86, 87, 88]. Notably, the parallel/antiparallel ratio increased most rapidly at 1200 rpm (Figure 6c), confirming that intense HSFs accelerate antiparallel‐to‐parallel rearrangement during fibril maturation (Figure 6e). Collectively, these results indicate that HSFs perturb β‐strand alignment kinetics and promote β‐sheet remodeling via inter‐sheet detachment–annealing or intra‐sheet reptation mechanisms [89], thereby facilitating structural stabilization. To corroborate cross‐β structure formation, Thioflavin T (Th T) fluorescence assays were performed (Figure 6d). The initially dissolved soy protein exhibited weak but detectable fluorescence, likely due to acid‐induced exposure of binding sites. Upon 6 h of acidic heating, fluorescence intensities increased by several orders of magnitude, with higher rotational speeds producing faster amplification. At 600 and 1200 rpm, maximal fluorescence was achieved at 6 h, whereas static, 200, and 400 rpm conditions required more than 12 h. These results demonstrate that shear flow fields can accelerate cross‐β structure formation. The fibril conversion rate also confirms this acceleration trend, attaining a maximum value of 82.33%±6% at 200 rpm, and it decreases remarkably under higher rotational speeds (Figure S16).

**FIGURE 6:**
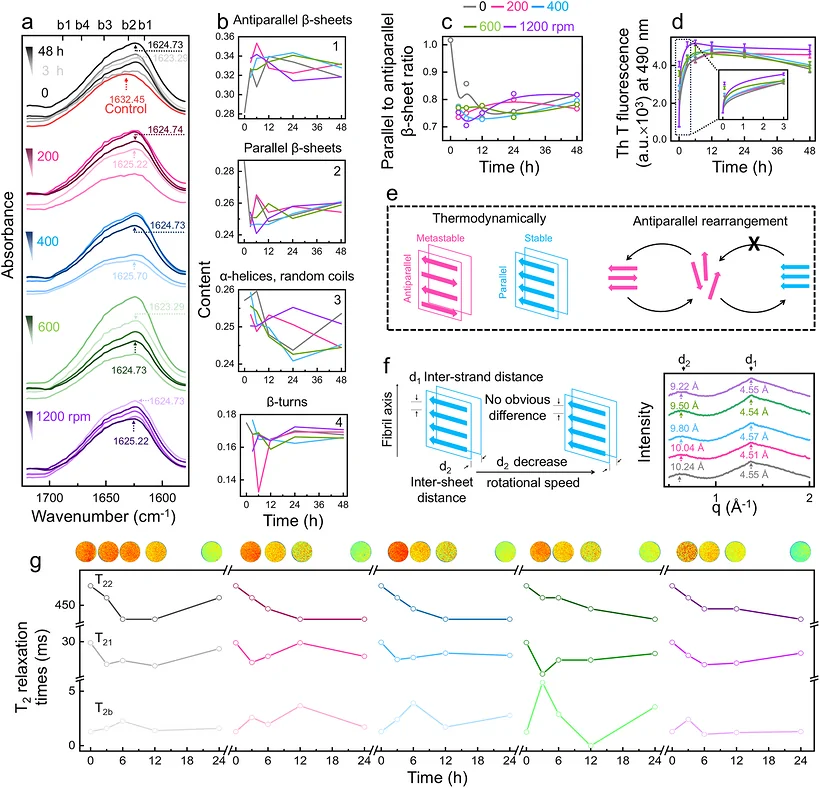
Conformational remodeling of SAFs. (a) The Amide I region (1600–1700 cm^−1^) of FTIR spectra of various soy protein samples obtained under different flow conditions. (b) The temporal evolution of secondary structures. (b1) antiparallel β‐sheets. (b2) parallel β‐sheets. (b3) α‐helices and/or random coils. (b4) β‐turns. (c) The changes in the ratios of parallel to antiparallel β‐sheet under various flow conditions. (d) The changes in Th T fluorescence intensity at emission wavelength of 490 nm under various flow conditions. (e) Shear flow fields accelerate antiparallel‐to‐parallel rearrangement during fibril maturation (f) The WAXS results probe atomistic rearrangements in SAFs after 48 h of heating. (g) The changes in spin‐spin relaxation time (T_2_) as a function of acidic heating time under various flow conditions.

Wide‐angle x‐ray scattering (WAXS) was further employed to probe atomistic rearrangements in SAFs after 48 h of heating (Figure 6f). Characteristic scattering peaks at 2π/4.5 Å^−1^ and 2π/10 Å^−1^ correspond to inter‐strand spacing (d_1_ ∼4.5 Å) and inter‐sheet spacing (d_2_ ∼10 Å), respectively [86, 90, 91]. While d_1_ remained nearly invariant across shear conditions, indicating preserved hydrogen bonding between adjacent β‐strands, d_2_ decreased markedly from 10.24 Å (static) to 9.22 Å (1200 rpm). This reduction in inter‐sheet distance indicates enhanced steric zipper interactions between adjacent β‐sheets under elevated HSFs, driving tighter side‐chain interdigitation and formation of a more energetically favorable cross‐β packing arrangement. To elucidate the differences in peptide composition and packing behavior, LC‐MS was further performed on hydrolysates collected from samples stirred at 0, 200, 600, and 1200 rpm after 6, 12, 24, and 48 h of acidic heating. The total ion chromatograms varied with heating time and rotational speed, indicating shear‐dependent peptide profiles (Figure S17a). To further characterize these differences, the numbers of unique peptides and peptide length distributions were quantified (Figure S17b,c). After 48 h, the 0, 200, 600, and 1200 rpm groups contained 4237, 4802, 4786, and 4594 unique peptides, respectively, corresponding to 357–565 additional peptides relative to the static condition. Peptide length distributions exhibited a distinct shear dependence. Compared with the static group, the proportion of short peptides (≤10 amino acids) increased from 30.68% to 32.26%, 33.41%, and 32.74% under 200, 600, and 1200 rpm, respectively, whereas the proportion of long peptides (> 30 amino acids) decreased from 5.97% to 4.46%, 3.84%, and 4.53%. Notably, the 600 rpm group exhibited the highest proportion of short peptides and the lowest proportion of long peptides, indicating enhanced peptide fragmentation under intermediate shear conditions. To further resolve flow‐dependent differences in peptide composition, peptides identified after 48 h were analyzed using an UpSet plot and abundance heatmap (Figure S18a,b). Although 2222 peptides were shared among all groups, consistent with a common acid hydrolysis pathway, each shear condition generated distinct peptide subsets, with 605, 609, 818, and 449 group‐specific peptides identified at 0, 200, 600, and 1200 rpm, respectively. The largest number of group‐specific peptides at 600 rpm indicates that shear flow altered peptide composition rather than merely increasing peptide abundance. Consistently, the heatmap of the 20 most abundant peptides revealed distinct abundance patterns, with several dominant peptides enriched at 600 rpm but generally reduced abundances at 1200 rpm. Interestingly, although 200 rpm produced the most uniform fibrils, it did not exhibit the highest degree of peptide fragmentation or compositional heterogeneity. Instead, this condition generated the largest overall peptide repertoire while maintaining fewer short peptides and fewer group‐specific peptides than the 600 rpm group. These results indicate that moderate shear stress facilitates a balanced hydrolysis state, which generates sufficient peptide diversity without inducing excessive fragmentation. Additionally, while these datasets do not directly reflect the peptide composition of the fibrillar fraction, the results indicate that different hydrodynamic conditions produce distinct soluble hydrolysis‐derived peptide profiles, which may be correlated with variations in subsequent fibril assembly processes. Thus, the reduction in inter‐sheet distance may be related to flow‐dependent alterations in peptide length distribution and dominant peptide abundance patterns, which can modify hydrogen bonding, hydrophobic interactions, steric effects, and β‐sheet stacking efficiency.

Given that steric zipper formation is accompanied by expulsion of interfacial water molecules [92, 93, 94], low‐field NMR (LF‐NMR) and MRI were performed to monitor water state transitions during fibrillation (Figure 6g and Figure S19). Three water populations were identified: peptide‐bound water (T_2b_, 1–10 ms), inter‐aggregate immobilized water (T_21_, 10–40 ms), and bulk‐like free water (T_22_, 400–600 ms) [95, 96]. During fibrillation, T_2b_ relaxation times under intermediate rotational speeds (200–600 rpm) fluctuated more strongly than under static or high shear (1200 rpm), suggesting dynamic rearrangement of hydrogen bonding networks at moderate shear flow fields. In contrast, both static and high shear conditions appeared to stabilize hydrogen bonding environments. After 12 h of heating under static and high shear conditions (600 and 1200 rpm), T_21_ relaxation times increased progressively, indicating enhanced water immobilization due to aggregate entanglement or amorphous cluster formation. Meanwhile, T_22_ relaxation times decreased more steeply at higher shear rates, reflecting more efficient dehydration [97, 98] or dewetting transitions [99]. Interestingly, under static conditions, T_22_ increased after 12 h, consistent with the presence of shorter fibrils or loosely packed aggregates incapable of restricting bulk water mobility. Taken together, the FTIR, Th T, WAXS, LC‐MS, and LF‐NMR analyses collectively demonstrate that shear flow fields promote conformational remodeling from metastable antiparallel β‐sheets to energetically stabilized parallel cross‐β architectures. This transition is accompanied by inter‐sheet compaction and enhanced dehydration, ultimately yielding SAFs with reduced inter‐sheet spacing and thermodynamically favorable steric zipper packing.

## 3 Conclusions

Amyloid fibrillation of plant proteins has traditionally been interpreted as a thermally driven self‐assembly process in which agitation primarily accelerates unfolding and shortens the lag phase. The present findings revise this paradigm by demonstrating that shear flow fields act as active regulators of structural selection that couple nucleation hierarchy, β‐sheet remodeling, and dehydration‐driven packing into a unified energy dissipation framework. Rather than uniformly enhancing fibrillation kinetics, shear flow fields redistribute kinetic partitioning among competing aggregation routes, suppressing locally entangled or amorphous pathways while promoting directed elongation and controlled protofilament organization. Under static or weakly convective conditions, heterogeneous nucleation events coexist, enabling accumulation of metastable antiparallel β‐sheet–rich intermediates and yielding structurally compliant or irregular polymorphs. In contrast, a stable linear straining shear regime imposes directional alignment and collision bias without inducing turbulent fragmentation, thereby guiding assembly toward structurally convergent fibrils characterized by reduced periodicity and enhanced rigidity. At the molecular scale, shear perturbs β‐strand alignment kinetics, accelerating the transition from metastable antiparallel β‐sheet configurations to thermodynamically favored parallel cross‐β architectures. This conformational remodeling is accompanied by selective inter‐sheet compaction, as evidenced by reduced inter‐sheet spacing while preserving inter‐strand hydrogen bonding distances, indicating that shear flow fields primarily reinforce steric zipper–like side‐chain interdigitation rather than altering backbone hydrogen bonding geometry. Concomitantly, low‐field NMR analyses reveal accelerated dehydration and dewetting transitions under shear, consistent with enhanced expulsion of interfacial water molecules that stabilize tightly packed cross‐β structures. These observations collectively support a shear‐coupled conformational compaction mechanism in which shear flow fields (i) bias nucleation interactions, (ii) accelerate β‐sheet realignment, and (iii) promote dehydration‐mediated inter‐sheet tightening, ultimately stabilizing energetically minimized SAFs polymorphs. Importantly, excessive shear disrupts laminar alignment and partially compromises structural regulation, underscoring the existence of an optimal mechanical window for polymorph selection, particularly in the laminar straining regime. Together, this multiscale integration establishes hydrodynamic shear not as a passive accelerator but as a governing physical parameter that modulates amyloid energy landscapes through coupled conformational and hydration remodeling. This hydrodynamic selection principle may extend to other protein‐based amyloid systems and informs the mechanical regulation of functional biomaterials.

## 4 Experimental Methods

### 4.1 Materials

Mature soybeans (Dongnong‐252), possessing a moisture content of 11%, were sourced from the College of Agriculture, Northeast Agricultural University (Harbin, China). From Sigma–Aldrich Ltd. (St. Louis, MO, USA), we obtained Thioflavin T (Th T) and 1‐aniline‐naphthalene‐8‐sulfonate (ANS). All remaining chemicals and solvents utilized in this study were of analytical grade.

### 4.2 Preparation of soy Protein Isolate

Soy protein isolate (SPI) is prepared based on the isoelectric precipitation method described in previous work [25]. In brief, milled soy flour underwent five defatting cycles using n‐hexane, followed by drying at ambient temperature. The defatted flour was then suspended in DI water (10:1 ratio), stirred gently for 2 h at room temperature, and the pH adjusted to 8.0 using 1 m NaOH. Next, the mixture was centrifuged (Centrifuge 3–15 K, Sigma) at 10 000×g and 4°C for 30 min. The supernatant was acidified to pH 4.5 with 1 m HCl and centrifuged again at 5000×g and 4°C for 15 min. The resulting precipitate was washed twice with DI water, with the suspension centrifuged after each washing step. Finally, the purified precipitate was resuspended in DI water, adjusted to pH 7.0, dialyzed against DI water (3500 Da bag, Solarbio, Beijing, China) for 24 h (4°C), and lyophilized. SPI with protein content of 90.52% were predominantly soy storage proteins, encompassing 7S globulin and 11S globulin subunits. Specifically, the major 7S globulin proteins consisted of α, α′, and β subunits, with sequence coverages ranging from 64.69% to 83.83% and 130–539 identified peptides. The major 11S globulin proteins included G1, G2, G3, G4, and G5 subunits, with sequence coverages ranging from 68.22% to 91.92% and 95–566 identified peptides (Table S2).

### 4.3 Fibrillation of SPI Under Different Flow Conditions

SPI powder (90.52% of protein) was used to create fibril‐forming solutions by dissolving it in DI water. The pH of fibril‐forming solutions was adjusted to pH 2.0 with 6 m HCl and then clarified via centrifugation (Centrifuge 3–15K, Sigma) at 10 000×g and 4°C for 30 min. With a protein concentration of 30 mg/mL, the solutions were heated to 85°C under magnetic stirring in a beaker, rotating at 0–1200 rpm for durations ranging from 0 to 48 h. All samples were subjected to pH stabilization treatment at a uniform time (10 min) during the initial heating phase, ensuring identical initial protein concentration, composition, and pH value. The corresponding samples were taken out at various time points during the heat treatment and stored at 4°C for later analysis.

### 4.4 Rheological Measurements

The oscillatory rheological experiments were performed using a shear rheometer (Discovery HR‐1, TA Instruments, New Castle, USA) at 25°C. The shear viscosity was evaluated across a shear rate gradient of 0.1 to 100 s^−1^. Frequency sweep rheology test was measured at 0.1% strain and the angular frequency was between 0.1 and 100 rad s^−1^.

### 4.5 Atomic Force Microscopy

Fibril‐forming solutions (0.01 mg/mL, pH 2.0) were applied to freshly cleaved mica surfaces (ca. 1.0 × 1.0 cm^2^) and allowed to air dry prior to atomic force microscopy (AFM) imaging. A Bruker dimension fast scan microscope, operating in tapping mode (Bruker Corp., Karlsruhe, Germany), was used to acquire the AFM scans. Data processing and statistical analysis were subsequently carried out using the Nanoscope analysis 1.5 software and FiberApp v2.1 software [30]. For each shear flow condition, at least 3 independently prepared samples were examined, and 10 AFM maps (3×3 µm) were acquired from different areas of each sample. Only clearly resolved and isolated fibrils were included in the analysis to avoid overlap‐induced measurement errors.

### 4.6 Transmission Electron Microscopy

Transmission electron microscopy (TEM) (Hitachi HT7800, Tokyo, Japan) was used to observe the morphology of protein samples, which were negatively stained with 2% uranyl acetate. Micrographs were captured with the Hitachi TEM system, operating at an accelerating voltage of 100 kV.

### 4.7 Th T Fluorescence Analysis

Th T stock solution was prepared by dissolving 8 mg Th T in 10 mL phosphate buffer (10 mm, pH 7.0, 150 mm NaCl), followed by filtration through a 0.22 µm syringe filter. Fluorescence intensity was measured using a fluorescence spectrophotometer (Hitachi, Japan) at an excitation wavelength of 460 nm and an emission wavelength from 470 to 600 nm. Background fluorescence from the Th T working solution was subtracted [100].

### 4.8 Nuclear Magnetic Resonance (NMR) Relaxometry and Magnetic Resonance Imaging (MRI)

Transverse relaxation times (T_2_) were determined using a pulsed NMR analyzer (Bruker Optik GmbH Ettlingen, Germany). Samples (10 mL) were placed centrally within the RF coil. The Carr‐Purcell‐Meiboom‐Gill (CPMG) sequence was employed for T_2_ measurement, with the following parameters: 16 scans, 10 000 echoes, an echo sampling frequency of 200 kHz, and a scan interval of 1.5 s. The relaxation profiles and T_2_ values were obtained by inverting the CPMG decay curves using dedicated T_2_ inversion software. Internal water distribution within the gels was visualized using MRI on a 21 MHz analyzer (Niumag Co., Ltd., Shanghai, China). Based on the CPMG‐derived T_2_ values, the imaging parameters were set to: repetition time (TR) of 500 ms, echo time (TE) of 0.2 ms, signal reception bandwidth (SW) of 200 kHz, and number of signal averages (NS) of 4. The resulting hydrogen proton images exhibited brightness levels proportional to the total water content.

### 4.9 Fourier‐Transform Infrared (FTIR) Spectroscopy

Lyophilized protein sample FTIR spectra were acquired using a Nicolet Magna 750 spectrometer (Nicolet, Madison, USA). The instrument scanned the range of 4000–400 cm^−1^ at a 4 cm^−1^ resolution, averaging 32 scans per sample. Secondary structure of the samples was analyzed within the amide I region (1700–1600 cm^−1^) using the Omnic 8 software and the PeakFit Version 4.12 software. Self‐deconvolution and peak fitting were performed to estimate the positions and relative areas of the underlying component bands, from which the relative secondary structure contents were calculated. Self‐deconvolution resolved five secondary structural components: antiparallel β‐sheets (1617 and 1680 cm^−1^), parallel β‐sheets (1632 cm^−1^), α‐helices/random coils (1648 cm^−1^), and β‐turns (1663 cm^−1^).

### 4.10 Wide‐Angle X‐Ray Scattering Measurements

Wide‐angle x‐ray scattering (WAXS) measurements were carried out at room temperature using the Xeuss instrument (Xenocs, Sassenage, France). The x‐ray source consisted of monochromatic Cu Kα radiation (λ = 1.54 Å), which was detected by a Rayonix SX165 CCD detector (Rayonix LLC, Evanston, IL, USA). The scattering range employed was 0.2–2.0 Å^−1^, and the scattering vector q was analyzed according to Equation (1):

(1) $$q=\frac{4\pi }{\lambda }\;\sin\frac{\theta }{2}$$

where q denotes the scattering vector, λ represents the wavelength of the incident radiation, and θ refers to the scattering angle. The formula used to calculate the interplanar distance (d) corresponding to the observed Bragg peaks. The scattering intensity measured through the freeze‐dried fibril samples was subsequently corrected.

### 4.11 CFD Simulation

To investigate the flow behavior, three‐dimensional transient CFD simulations were performed using the Navier–Stokes equations. The volume‐of‐fluid (VOF) method was employed to capture the free surface [101], while the Lagrangian particle‐tracking model was used to simulate the motion of the protein particles. The governing equations for the fluid consisted of the Continuity equation, Momentum equation, and Energy conservation equation, given as Equations (2)–(4), respectively.

(2) $$\frac{\partial \rho }{\partial t}+\frac{\partial }{\partial x_{i}}\left(\rho u_{i}\right)=0$$

(3) $$\frac{\partial }{\partial t}\left(\rho u_{i}\right)+\frac{\partial }{\partial x_{j}}\;\left(\rho u_{i}u_{j}\right)=-\frac{\partial p}{\partial x_{i}}+\frac{\partial \tau_{ij}}{\partial x_{j}}+\rho g_{i}+F_{i}$$

(4) $$\frac{\partial }{\partial t}\left(\rho h\right)+\frac{\partial }{\partial x_{i}}\;\left(\rho u_{i}h\right)=\frac{\partial }{\partial x_{i}}\;\left(k+k_{t}\right)\frac{\partial T}{\partial x_{i}}+S_{h}$$

where *ρ* was the fluid density, *𝑢_i_* was the component of fluid velocity in the i‐direction, *p* was the static pressure, *τ_ij_* was the ij‐component of the viscous stress tensor, *ρg_i_* was the gravitational force component in the i‐direction, *F_i_* was the other momentum source terms, ℎ was the specific enthalpy of the fluid, *k* was the molecular thermal conductivity, *k_t_* was the thermal conductivity associated with turbulent transport, *S_ℎ_* was the volumetric energy source term. All governing equations were solved using ANSYS Fluent 6.4 (Ansys Inc., USA). The CFD simulations were performed until a statistically steady flow field was obtained, without assuming any direct temporal scaling between the CFD simulation time and the experimental fibrillation time. Experimentally measured apparent viscosities at selected fibrillation stages were imported into the CFD software in tabulated form and assigned as the liquid‐phase dynamic viscosity. A separate CFD case was used for each stage, with the corresponding viscosity held constant during the simulation. The viscosity was measured at 25°C, and no temperature‐dependent viscosity model or correction to the fibrillation temperature of 85°C was applied. Specifically, we used a cylindrical beaker with a screw cap, having an outer diameter of 35 mm, a height of 55 mm, and a wall thickness of 1 mm. The solution height in the beaker was kept at 25 mm. A magnetic stir bar (6 mm in diameter × 25 mm in length) was employed for agitation. The schematic diagram of the solution model and model grid were shown in Figure S20a. At different rotational speeds, a constant heat source of 85°C was applied at the bottom, an external temperature of 20°C was applied at the top, and a convective heat transfer of 20°C was applied to the wall surface. The comprehensive convective heat transfer coefficient was taken as 1 W/(m^2^·K), and the equivalent thermal resistance of the wall surface was taken as 0.001 (m^2^·K)/W. The wall treatment adopted the full *y+* wall treatment approach, which employed a mixed wall function. For fine grids, it emulated the low *y+* wall treatment, while for coarse grids, it emulated the high *y+* wall treatment (Figure S20b). Because of limitations in computational resources, no systematic mesh‐independence analysis was performed, and no quantitative CFD–PIV comparison was conducted. Therefore, the CFD results were used only as complementary hydrodynamic support for the proposed mechanism, rather than as quantitative validation of the flow field or fibrillation pathway.

### 4.12 PIV Experiments

The PIV experiments mainly track the tiny titanium dioxide particles scattered in the flow field through image analysis technology and characterize the fluid motion based on the movement of these particles. It is equipped with a high‐speed camera with a maximum full‐frame frame rate of 3000 fps (Qianyanlang M230, China), as well as a 10 W continuous laser with a wavelength of 532 nm. The two‐dimensional flow velocity vectors at each point in the plane were measured using a planar two‐dimensional high‐frequency particle image velocimetry system (2D2C‐PIV) in both time series mode (1–10 KHz) and cross‐frame mode (≥800 Hz).

### 4.13 Fibril Conversion Rate Measurements

The fibril conversion rate was determined following the method of Akkermans et al., with minor modifications [102]. Fibril‐forming solutions prepared under different treatment conditions were diluted to 1.0 mg/mL and subjected to centrifugal ultrafiltration using 100 kDa cutoff membranes. Upon centrifugation, fibrillar aggregates were retained by the membrane, while unassembled proteins and soluble peptides passed into the filtrate. Total protein content prior to ultrafiltration and protein content in the filtrate were quantified using the Lowry assay. Fibril conversion rate was calculated as the difference between the initial total protein content and the unassembled protein fraction in the filtrate, normalized to the initial total protein content.

### 4.14 Liquid Chromatography‐Tandem Mass Spectrometry (LC‐MS) Analysis

Hydrolysis products collected under various flow conditions (0, 200, 600, and 1200 rpm) at different incubation durations (6, 12, 24, and 48 h) were centrifuged to eliminate insoluble materials. Subsequently, the supernatants were filtered through a 0.22 µm membrane prior to LC‐MS analysis. Peptide separation was conducted using a Thermo Vanquish UHPLC system coupled with an Orbitrap Exploris mass spectrometer (Thermo Fisher Scientific, Bremen, Germany). The separation was carried out on a reversed‐phase C18 column with mobile phase A, which was 0.1% formic acid in water, and mobile phase B, which was 0.1% formic acid in acetonitrile, at a flow rate of 0.40 mL/min. The gradient was as follows: 8% B from 0 to 4 min, 8%–35% B from 4 to 48 min, 35%–99% B from 48 to 51 min, 99% B from 51 to 54 min, followed by re‐equilibration at 8% B until 60 min. Mass spectrometric analysis was performed in the positive ion mode. Data were acquired by means of a data‐dependent acquisition (DDA) method with ddMS^2^ fragmentation. Full MS scans were obtained over an m/z range of 350–1500 at a resolution of 60 000, with an AGC target of 300%, a maximum injection time of 50 ms, and an RF lens setting of 40%. Precursor ions with charge states of 2–6 were chosen for higher‐energy collisional dissociation (HCD). MS spectra were acquired at a resolution of 15 000 using a normalized collision energy of 30%, an isolation window of 1.6 m/z, a first mass of m/z 120, an AGC target of 75%, and a maximum injection time of 30 ms. Dynamic exclusion was set at 30 s, and precursor ions were selected within a 2 s cycle time. The ion transfer tube temperature was 320°C, and the spray voltage was 2.0 kV. Raw data were processed using Proteome Discoverer 3.0 and subjected to a search against the UniProt Glycine max protein database. Given that the peptides were generated through acid‐heating‐induced hydrolysis instead of enzymatic digestion, database searching was carried out without specifying an enzyme. The precursor and fragment mass tolerances were respectively set to 15 ppm and 0.02 Da. Peptide identification was filtered at a false discovery rate (FDR) of less than 1% [103]. Non‐redundant peptide sequences were employed for peptide composition analysis, which included the determination of peptide number, length distribution, overlap analysis, and assessment of dominant peptide abundance.

### 4.15 Statistical Analysis

All measurements were taken in triplicate, with results presented as mean ± standard deviation (SD). A one‐way analysis of variance (ANOVA) assessed the significance of main effects, and multiple comparisons were conducted using Duncan's test, establishing significance at *p* < 0.05. Statistical analysis utilized SPSS software (SPSS Inc., Evanston, IL, USA).

## Author Contributions

**Zejian Xu**: conceptualization, investigation, methodology, writing – original draft, software, data curation. **Pengfei Yang**: methodology. **Meng Li**: methodology. **Zichen Cao**: methodology. **Tian Lan**: methodology. **Yan Zhang**: investigation. **Weibiao Zhou**: investigation. **Lianzhou Jiang**: investigation. **Xiaonan Sui**: conceptualization, investigation, supervision, writing – review and editing, project administration.

## Funding

Distinguished Young Scientists Fund of National Natural Science Foundation of China (No.: 32325041), Key Program of National Natural Science Foundation of China (No.: 32230082).

## Conflicts of Interest

The authors declare no conflicts of interest.

## Supporting information




**Supporting File 1**: advs78458‐sup‐0001‐SuppMat.docx.

**Supporting File 2**: advs78458‐sup‐0002‐MovieS1.mp4.

**Supporting File 3**: advs78458‐sup‐0003‐MovieS2.mp4.

**Supporting File 4**: advs78458‐sup‐0004‐MovieS3.mp4.

**Supporting File 5**: advs78458‐sup‐0005‐MovieS4.mp4.

**Supporting File 6**: advs78458‐sup‐0006‐MovieS5.mp4.

**Supporting File 7**: advs78458‐sup‐0007‐MovieS6.mp4.

**Supporting File 8**: advs78458‐sup‐0008‐MovieS7.mp4.

**Supporting File 9**: advs78458‐sup‐0009‐MovieS8.mp4.

**Supporting File 10**: advs78458‐sup‐0010‐MovieS9.mp4.

**Supporting File 11**: advs78458‐sup‐0011‐MovieS10.mp4.

**Supporting File 12**: advs78458‐sup‐0012‐MovieS11.mp4.

**Supporting File 13**: advs78458‐sup‐0013‐MovieS12.mp4.

**Supporting File 14**: advs78458‐sup‐0014‐MovieS13.mp4.

**Supporting File 15**: advs78458‐sup‐0015‐MovieS14.mp4.

**Supporting File 16**: advs78458‐sup‐0016‐MovieS15.mp4.

## Data Availability

The data that support the findings of this study are available on request from the corresponding author. The data are not publicly available due to privacy or ethical restrictions.
