# Supplementary material for: Hydrodynamic Selection of Polymorphic Assembly Pathways in Plant Protein Amyloid Fibrils
Source: Adv Sci (Weinh). 2026 Oct 26:e78458. Online ahead of print. doi: 10.1002/advs.78458 (PMC13710711; doi:10.1002/advs.78458)
Supplement: Supplementary file 1 [file ADVS-9999-e78458-s006.docx]

Hydrodynamic selection of polymorphic assembly pathways in plant protein amyloid fibrils

Zejian Xu, Pengfei Yang, Meng Li, Zichen Cao, Tian Lan, Yan Zhang, Weibiao Zhou, Lianzhou Jiang*, Xiaonan Sui*

Z. Xu, P. Yang, M. Li, Z. Cao, T. Lan, L. Jiang, X. Sui

College of Food Science, Northeast Agricultural University, Harbin 150030, P. R. China

Heilongjiang Joint Laboratory of Plant-Based Food Science (International Cooperation), Harbin 150030, P. R. China

X. Sui, E-mail: [xiaonan.sui@neau.edu.cn](mailto:xiaonan.sui@neau.edu.cn); L. Jiang, E-mail: [jlzname@163.com](mailto:jlzname@163.com)

W. Zhou

Department of Food Science and Technology, National University of Singapore, 2 Science Drive 2 117542, Singapore

Y. Zhang

College of Horticulture and Landscape Architecture, Northeast Agricultural University, Harbin 150030, P. R. China

Funding:

Distinguished Young Scientists Fund of National Natural Science Foundation of China (No.: 32325041)

Key Program of National Natural Science Foundation of China (No.: 32230082).

Keywords: plant protein, amyloid fibrils, hydrodynamic selection, polymorphic assembly pathways

**Supporting Information**

**Hydrodynamic selection of polymorphic assembly pathways in plant protein amyloid fibrils**

*Zejian Xu, Pengfei Yang, Meng Li, Zichen Cao, Tian Lan, Yan Zhang, Weibiao Zhou, Lianzhou Jiang*, Xiaonan Sui**

**Figure S1.** (a) The javelin-like fibrils (type 2). (b, c) The loop-like fibrils (type 3)

**Figure S2.** (a) The javelin-like fibrils (average heights of closing to 2 nm) with tapered ends and their fibril-elongation processes.^[1]^ (b) The ICL of helical ribbon fibrils (type 5) obtained at the rotational speed of 200 rpm.

**Figure S3.** AFM observations of the diluted fibril-forming solutions obtained at various shear flow conditions and acidic heating time of 6-48 h.

**Figure S4.** The height profiles along the longitudinal sections of targeted fibrils obtained at acidic heating time of (a) 12 and (b) 48 h (highlighted by side-to-side dots in **Figure S3**).

**Figure S5.** (a, b) The average height (less than 1 nm) and variability of periodicity of single-twisted ribbon fibrils obtained under static condition and (c, d) shear flow conditions.

**Figure S6.** The storage modulus (G’) and loss modulus (G’’) of fibril hydrogels obtained at (a) 200 rpm (*Re* ≈ 1345) and (b) 600 rpm (*Re* ≈ 8649) following acidic heating for 6, 12, and 24 hours measured at 0.1% strain and the angular frequency was between 0.1 and 100 rad s^-1^.

**Figure S7.** The average velocity cloud charts and velocity distribution statistics chart.

**Figure S8.** The contours of vertical and lateral (a) vorticity, (b) velocity, and (c) turbulent kinetic energy under static conditions obtained by CFD simulations.

**Figure S9.** The contours of vertical and lateral (a) vorticity, (b) velocity, and (c) turbulent kinetic energy at rotational speed of 200 rpm obtained by CFD simulations.

**Figure S10.** The contours of vertical and lateral (a) vorticity, (b) velocity, and (c) turbulent kinetic energy at rotational speed of 400 rpm obtained by CFD simulations.

**Figure S11.** The contours of vertical and lateral (a) vorticity, (b) velocity, and (c) turbulent kinetic energy at rotational speed of 600 rpm obtained by CFD simulations.

**Figure S12.** The contours of vertical and lateral (a) vorticity, (b) velocity, and (c) turbulent kinetic energy at rotational speed of 1200 rpm obtained by CFD simulations.

**Figure S13.** Spatial distributions of turbulent energy dissipation rate (ε, m^2^/s^3^) extracted from CFD simulations under different rotational speeds. Rows from top to bottom correspond to (a) 0, (b) 200, (c) 400, (d) 600, and (e) 1200 rpm, respectively. Columns from left to right correspond to representative simulation times of 5, 35, 65, 95, 125, and 175 s. Higher ε values indicate stronger local viscous dissipation and enhanced hydrodynamic forcing.

**Figure S14.** Spatial distributions of Kolmogorov length scale (η, m) extracted from CFD simulations under different rotational speeds. Rows from top to bottom correspond to (a) 0, (b) 200, (c) 400, (d) 600, and (e) 1200 rpm, respectively. Columns from left to right correspond to representative simulation times of 5, 35, 65, 95, 125, and 175 s. Smaller η values indicate smaller dissipative eddies and stronger local-scale hydrodynamic heterogeneity.

**Figure S15.** Spatial distributions of Kolmogorov time scale (τk, s) extracted from CFD simulations under different rotational speeds. Rows from top to bottom correspond to (a) 0, (b) 200, (c) 400, (d) 600, and (e) 1200 rpm, respectively. Columns from left to right correspond to representative simulation times of 5, 35, 65, 95, 125, and 175 s. Smaller τk values indicate shorter local deformation timescales and more rapid hydrodynamic perturbations.

**Figure S16.** Fibril conversion rate under different rotational speeds during the acidic heating period of 48 h.

**Figure S17.** LC-MS profiling and peptide length distribution of hydrolysis products under different flow conditions. (a) Total ion chromatograms (TICs) of peptides derived from the hydrolysis of SPI were obtained under different flow conditions (0, 200, 600, and 1200 rpm) during acidic heating at pH 2.0 and 85 °C for 6, 12, 24, and 48 h; (b) The number of unique peptides identified under different flow conditions and heating durations; (c) A heatmap depicting the peptide length distribution of hydrolysis-derived peptides. Peptides were classified into six length ranges: ≤ 10, 11-15, 16-20, 21-25, 26-30, and > 30 amino acids. The color scale indicates the percentage of unique peptides in each length range.

**Figure S18.** Composition of shared/unique peptides and dominant patterns of peptide abundance under different flow conditions at 48 h. (a) An UpSet plot is presented to illustrate the shared and unique peptide sequences identified within the obtained hydrolysis products. The presence of peptides was defined based on the non-redundant peptide sequences identified by LC-MS; (b) Relative abundance heatmap of the top 20 hydrolysis-derived peptides. The top 20 peptides were selected according to their total peak areas across the four groups. The color scale represents the row-wise Z-score of log10-transformed peptide area

**Figure S19.** T_2_ relaxation time distribution curves.

**Figure S20.** (a) The schematic diagram of the solution model and model grid. (b) The re-normalization group (RNG) k–ε model.

**Table S1.** Secondary structural contents of protein samples

| RP (rpm) | T (h) | Antiparallel β-sheet (%) | Parallel β-sheet (%) | β-sheet  (%) | α-helices and/or random coils (%) | β-turns  (%) | Parallel/antiparallel β-sheet ratio |
| --- | --- | --- | --- | --- | --- | --- | --- |
| 0 | 0  3  6  12  24 | 28.16 | 28.63 | 56.80 | 25.72 | 17.49 | 1.02 |
|  |  | 32.70 | 24.67 | 57.37 | 25.83 | 16.81 | 0.75 |
|  |  | 30.81 | 26.42 | 57.23 | 25.96 | 16.81 | 0.86 |
|  |  | 33.96 | 24.84 | 58.80 | 24.79 | 16.41 | 0.73 |
|  |  | 33.39 | 25.23 | 58.62 | 24.37 | 17.00 | 0.76 |
|  | 48 | 31.84 | 26.04 | 57.88 | 25.36 | 16.76 | 0.82 |
| 200 | 3 | 33.35 | 24.52 | 57.87 | 25.33 | 16.80 | 0.74 |
|  | 6 | 35.34 | 26.52 | 61.86 | 24.88 | 13.26 | 0.75 |
|  | 12 | 32.75 | 25.42 | 58.18 | 25.32 | 16.50 | 0.78 |
|  | 24 | 32.23 | 25.78 | 58.01 | 25.06 | 16.93 | 0.80 |
|  | 48 | 33.20 | 25.43 | 58.63 | 24.44 | 16.93 | 0.77 |
| 400 | 3 | 31.79 | 24.63 | 56.41 | 25.92 | 17.66 | 0.77 |
|  | 6 | 33.77 | 24.67 | 58.44 | 25.08 | 16.48 | 0.73 |
|  | 12 | 33.90 | 24.65 | 58.55 | 24.99 | 16.46 | 0.73 |
|  | 24 | 34.34 | 25.33 | 59.68 | 24.08 | 16.24 | 0.74 |
|  | 48 | 32.79 | 26.10 | 58.89 | 24.52 | 16.59 | 0.80 |
| 600 | 3 | 32.58 | 25.08 | 57.66 | 25.56 | 16.78 | 0.77 |
|  | 6 | 32.60 | 25.09 | 57.69 | 25.42 | 16.89 | 0.77 |
|  | 12 | 33.41 | 25.97 | 59.38 | 24.74 | 15.88 | 0.78 |
|  | 24 | 34.07 | 25.03 | 59.11 | 24.26 | 16.63 | 0.73 |
|  | 48 | 33.12 | 25.88 | 59.00 | 24.44 | 16.56 | 0.78 |
| 1200 | 3 | 33.79 | 25.42 | 59.21 | 25.03 | 15.76 | 0.75 |
|  | 6 | 34.17 | 24.09 | 58.25 | 25.02 | 16.73 | 0.70 |
|  | 12 | 33.35 | 25.04 | 58.39 | 25.22 | 16.39 | 0.75 |
|  | 24 | 31.43 | 25.80 | 57.23 | 25.52 | 17.24 | 0.82 |
|  | 48 | 31.83 | 26.01 | 57.85 | 25.08 | 17.07 | 0.82 |

**Table S2.** Major protein species identified by LC-MS in the SPI-derived samples

| Protein family | Major identified protein species | Sequence coverage (%) | No. of identified peptides |
| --- | --- | --- | --- |
| 7S  globulin | α, α′, and β subunits | 64.69-83.83 | 130-539 |
| 11S  globulin | G1, G2, G3, G4, and G5 subunits | 68.22-91.92 | 95-566 |

**References**

1. Z. Xu et al., “Structural insights into acidic heating-induced amyloid fibrils derived from soy protein as a function of protein concentration,” *Food Hydrocolloids* 145 (2023): 109085.
